# Agitation severity and emergency department outcomes in substance-related behavioral emergencies: a retrospective cohort study

**DOI:** 10.1186/s12873-026-01646-0

**Published:** 2026-06-18

**Authors:** Asli Bahar Ucar, Nurseli Bayram, Gizem Bozkurt, Mustafa Aytemiz, Ezgi Hilal Ozan, Arzu Denizbası Altinok

**Affiliations:** 1 Department of Emergency Medicine, Marmara University Pendik Training and Research Hospital, Muhsin Yazicioglu Cd. No: 10, Pendik, Istanbul, 34899 Türkiye; 2 Department of Emergency Medicine, Marmara University Faculty of Medicine, Muhsin Yazicioglu Cd. No: 10, Pendik, Istanbul, 34899 Türkiye

**Keywords:** Agitation, Benzodiazepines, Emergency department, Polysubstance use, Substance-related disorders, Overt agitation severity scale, OASS

## Abstract

**Background:**

Substance-related emergency department (ED) visits increasingly present with agitation and behavioral instability rather than discrete toxidromes. Early decisions regarding escalation and disposition may be influenced by observable behavioral severity in addition to suspected substance category and initial pharmacologic regimen. We evaluated predictors of pharmacologic escalation and psychiatric admission among adults requiring psychoactive medication for substance-related behavioral disturbance.

**Methods:**

This retrospective cohort study was conducted in a tertiary ED between January 2024 and January 2025. Adult patients with clinician-judged substance-related presentations who received antipsychotics or benzodiazepines for immediate behavioral control were included. Agitation severity was assessed using the Overt Agitation Severity Scale (OASS) and categorized as mild (0), moderate (1), or severe (≥ 2). The primary outcome was pharmacologic escalation, defined as the addition of a second antipsychotic or benzodiazepine. The secondary outcome was psychiatric admission. Predictors were evaluated using multivariable logistic regression. Model discrimination was assessed using bootstrap internal validation (1,000 resamples). An exploratory receiver operating characteristic (ROC) analysis was performed to evaluate the discriminative performance of OASS.

**Results:**

Ninety encounters were analyzed (median age 32 years; 86.7% male), of which 61.1% had severe agitation (OASS ≥ 2). Pharmacologic escalation occurred in 23.3% and psychiatric admission in 71.1% of cases. Both outcomes increased across OASS strata (*p* = 0.012 and *p* = 0.016, respectively). In multivariable analysis, severe agitation independently predicted psychiatric admission (adjusted OR 7.87, 95% CI 2.28–27.17), while increasing age was inversely associated (adjusted OR 0.91 per year, 95% CI 0.86–0.96). The optimism-corrected AUC for psychiatric admission was 0.76 (95% CI 0.66–0.85). In exploratory analysis, OASS ≥ 2 demonstrated a sensitivity of 81.8% and a negative likelihood ratio of 0.36.

**Conclusions:**

Agitation severity was independently associated with psychiatric admission and showed a graded association with pharmacologic escalation in unadjusted analyses across OASS strata. Structured behavioral assessment using OASS may support early risk stratification and disposition planning in substance-related agitation.

**Supplementary Information:**

The online version contains supplementary material available at https://doi.org/10.1186/s12873-026-01646-0.

## Introduction

Substance-related presentations constitute a major and growing operational challenge for emergency departments (EDs). Contemporary ED encounters increasingly involve behavioral instability—agitation, aggression, psychotic symptoms, and suicidality—rather than isolated, easily classifiable toxidromes [1–3]. This trend reflects both global patterns and regional increases in polysubstance use, particularly involving stimulants and synthetic cannabinoids [4, 5]. Polysubstance exposure, limited collateral information, and ED crowding frequently create diagnostic uncertainty, delay behavioral assessment, reduce staffing flexibility, and complicate safe management at the moment when immediate behavioral control is required to ensure safety and enable evaluation [6–8].

In clinical practice, clinicians may consider the suspected substance category (e.g., stimulant intoxication versus alcohol or opioid withdrawal) when selecting early management strategies. However, at the bedside the most urgent question is commonly not which substance, but rather the severity of the current behavioral disturbance, as this determines containment needs, staffing requirements, and the feasibility of diagnostic work-up. Antipsychotics and benzodiazepines are widely used as first-line agents for rapid stabilization [7–10], yet evidence guiding medication selection in substance-related behavioral emergencies remains largely extrapolated from psychiatric or withdrawal-focused cohorts rather than ED populations defined by acute behavioral risk.

Withdrawal instruments such as the Clinical Institute Withdrawal Assessment for Alcohol–Revised (CIWA-Ar) and the Clinical Opiate Withdrawal Scale (COWS) are indispensable for syndrome-specific monitoring, but they emphasize physiologic symptom clusters and are not consistently feasible in highly agitated patients [9, 11]. By contrast, brief observational agitation measures may better align with ED priorities because they quantify observable interference with care, verbal/physical aggression, and motor agitation—dimensions directly linked to safety and disposition. The Overt Agitation Severity Scale (OASS) was developed for objective rating of agitation and captures these domains in a format suited to urgent environments [12].

Despite this evidence, the emergency department literature remains heterogeneous. Previous ED studies have described the prevalence, characteristics, and management of agitation or compared pharmacologic strategies for behavioral control [8, 10, 13–15], but relatively few have examined whether short-term outcomes are more closely related to substance attribution or observable agitation severity.

Although various agitation scales are available, most were developed for psychiatric or inpatient settings rather than emergency departments [12, 16]. In the ED, agitation is commonly assessed through bedside clinical judgment, especially when information is limited and rapid decisions are required. Structured scales such as OASS are therefore not routinely used during early escalation or disposition decisions. Whether these tools can improve early ED decision-making in substance-related behavioral emergencies remains unclear.

To address this gap, we conducted a one-year retrospective cohort study of adults presenting to a tertiary ED with substance-related behavioral disturbances requiring psychoactive medication. Our objectives were to (i) characterize real-world pharmacologic strategies, (ii) examine the relationship between agitation severity and pharmacologic escalation, and (iii) identify factors associated with psychiatric admission. We hypothesized that agitation severity would show closer associations with pharmacologic escalation and psychiatric admission than substance category or initial medication class.

## Methods

### Study design and setting

This retrospective observational cohort study was conducted in the adult emergency department of Marmara University Pendik Training and Research Hospital, a tertiary university-affiliated center in Istanbul, Türkiye, with an annual census exceeding 200,000 visits and 24-hour psychiatric consultation availability. The study was conducted between 1 January 2024 and 1 January 2025. The study protocol was approved by Marmara University Faculty of Medicine Clinical Research Ethics Committee (Approval No. 09.2025.25–0496). The requirement for informed consent was waived due to the retrospective design and the use of de-identified clinical data. This study adheres to the Strengthening the Reporting of Observational Studies in Epidemiology (STROBE) guidelines for cohort studies [17]. A completed STROBE checklist was submitted separately.

### Study population

Adult patients (≥ 18 years) were eligible if their ED presentation was judged by the treating physician to be substance-related and if they received at least one psychoactive medication for immediate behavioral control during the index visit. Substance classification relied on clinical judgment (history, examination findings, treatment response) without routine toxicology testing. Encounters were excluded when the final diagnosis reflected a non-psychiatric medical etiology explaining the behavioral disturbance (e.g., hypoglycemia, electrolyte imbalance, central nervous system infection, intracranial hemorrhage), when documentation was insufficient to assign an Overt Agitation Severity Scale (OASS) score, or when the administered medication class or dose could not be reliably abstracted. To maintain statistical independence, only the first eligible encounter per patient within the study window was included.

### Behavioral assessment and OASS operationalization

Agitation severity was evaluated using the Overt Agitation Severity Scale (OASS), which rates four observable domains—motor activity, verbal aggression, physical aggression, and interference with care—each on a 0–2 scale (total range 0–6). For primary analyses, OASS was categorized as mild (0), moderate (1), and severe (≥ 2). The ≥ 2 threshold was selected a priori in accordance with the original OASS framework [12], where a score of 2 corresponds to the presence of physical aggression or marked interference with care requiring immediate intervention, representing the operational ED definition of severe agitation. We additionally explored discrimination using raw OASS scores and ROC analysis with Youden-derived cut-points.

### Pharmacologic variables and definition of escalation

All psychoactive medications administered for behavioral control were recorded, including agent, route, and cumulative dose when available. Drugs were grouped as antipsychotics (haloperidol, risperidone, olanzapine, chlorpromazine) or benzodiazepines (diazepam, midazolam). Biperiden was considered an adjunctive anticholinergic agent rather than a primary behavioral treatment.

The initial regimen was defined as the first psychoactive medication administered, excluding adjunctive agents. Pharmacologic escalation was defined as administration of ≥ 1 additional non-adjunctive psychoactive agent (antipsychotic or benzodiazepine) after the initial regimen for persistent or recurrent behavioral disturbance during the same ED encounter. Use of biperiden alone was not considered escalation, as it addresses extrapyramidal symptoms rather than behavioral control. Dose escalation of the same pharmacologic agent was not considered pharmacologic escalation, as it represents dose titration rather than a change in treatment strategy. This definition was intended to capture clinically meaningful treatment intensification rather than routine dose adjustment.

### Withdrawal measures

When documented as part of routine care, alcohol withdrawal severity was assessed using CIWA-Ar and opioid withdrawal using COWS. Because these instruments were not applied universally and primarily guided syndrome-specific management rather than immediate behavioral containment, they were analyzed descriptively within applicable subgroups and were not entered into the primary multivariable model.

### Study outcomes

The primary outcome was pharmacologic escalation. The secondary outcome was psychiatric admission from the ED (vs. discharge). Psychiatric admission was chosen as the disposition endpoint because it directly reflects safety assessment, resource utilization, and ED operational flow in behavioral emergencies.

### Data abstraction

Potentially eligible encounters were identified through ED diagnostic and disposition codes related to substance use and behavioral disturbance. Three trained abstractors independently reviewed electronic records using a standardized abstraction form. Inter-rater reliability for OASS scoring was high (κ = 0.82, 95% CI 0.75–0.89), assessed on a random sample of 30 encounters. OASS scores were retrospectively assigned based on documented behavioral descriptors in the electronic medical record. Disagreements were resolved by consensus, and all data were de-identified prior to analysis.

### Statistical analysis

Categorical and continuous variables were summarized using standard descriptive statistics. Group comparisons used χ² or Fisher’s exact tests and Mann–Whitney U tests as appropriate. Trend across OASS strata was assessed using the Cochran-Armitage test. Predictors of psychiatric admission were examined using multivariable logistic regression. Variables with *p* < 0.20 in univariate analysis (age, OASS severity, physical aggression, stimulant involvement) and clinically relevant covariates identified a priori (initial regimen) were considered for inclusion. Multicollinearity was assessed using variance inflation factors; all VIF values were < 5 (maximum 2.1). Model calibration was assessed with the Hosmer–Lemeshow test and overall fit with the likelihood ratio statistic. A two-sided *p* < 0.05 was considered statistically significant.

Discrimination for psychiatric admission was assessed using the area under the receiver operating characteristic curve (AUC). To limit optimism associated with model performance in this modest cohort, we performed bootstrap internal validation with 1,000 resamples and report the optimism-corrected AUC with 95% confidence intervals.

An exploratory receiver operating characteristic (ROC) analysis was conducted to identify a pragmatic OASS threshold for psychiatric admission using the Youden index. This analysis was intended to enhance clinical interpretability rather than establish a definitive diagnostic threshold.

All statistical analyses were performed using jamovi version 2.4 [18], an open-source statistical platform built on R. Given the modest sample size, model interpretation focused primarily on effect sizes, confidence intervals, and clinical plausibility rather than statistical significance alone. No imputation was performed for missing data. Encounters with insufficient documentation for key study variables were excluded from analysis.

### Exploratory post-hoc power estimate

Because the study was retrospective with a fixed observation window, no prospective sample size calculation was performed and the analytic sample was determined by the number of eligible encounters. An exploratory post-hoc estimate based on the observed difference in psychiatric admission between severe and non-severe agitation suggested 86% power at α = 0.05; however, interpretation focused primarily on effect sizes and confidence intervals rather than hypothesis testing alone.

## Results

### Study population

**Fig. 1:**
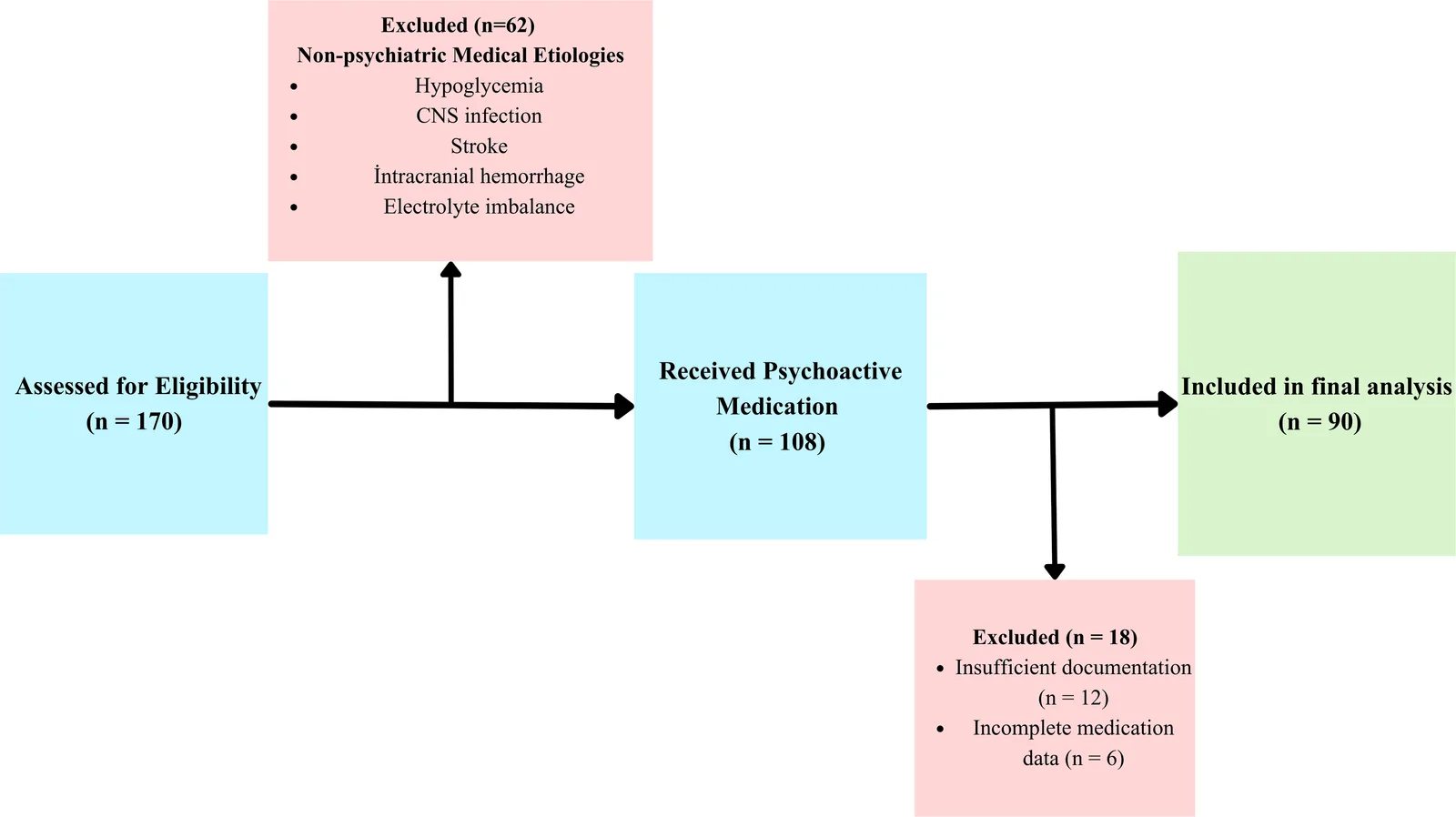
STROBE flow diagram of patient selection

During the 12-month period, 170 adult ED encounters received psychoactive pharmacologic treatment for acute behavioral disturbance. Sixty-two encounters were excluded because the final diagnosis reflected non-psychiatric medical etiologies mimicking agitation, including hypoglycemia (*n* = 25), electrolyte imbalance or severe metabolic derangement (*n* = 12), other acute medical conditions judged to be the primary cause of behavioral disturbance (*n* = 11), intracranial hemorrhage (*n* = 9), and central nervous system infection (*n* = 5). An additional 18 encounters were excluded due to insufficient documentation to classify agitation/withdrawal severity (*n* = 12) or incomplete pharmacologic data (*n* = 6). The final analytic cohort comprised 90 unique encounters (Fig. 1).

Median age was 32 years (IQR 25–39) and 86.7% were male. Primary documented substance category was polysubstance in 44.4%, stimulant only in 33.3%, alcohol only in 15.6%, and opioid only in 6.7%. Considering “any involvement,” stimulants were present in 77.8%, alcohol in 60.0%, and opioids in 51.1%, reflecting frequent combinations **(**Table 1**).**

**Table 1:** Baseline characteristics of adults with substance-related behavioral disturbance

Variable	Value
**Age**,** years**,** median (IQR)**	32 (25–39)
**Sex**,** n (%)**	
Male	78 (86.7)
Female	12 (13.3)
**Primary documented substance category**,** n (%)**	
Alcohol only	14 (15.6)
Opioid only	6 (6.7)
Stimulant only	30 (33.3)
Polysubstance (≥ 2 substances)	40 (44.4)
*Any substance involvement*,* n (%)* *	
Any alcohol	54 (60.0)
Any opioid	46 (51.1)
Any stimulant	70 (77.8)
**Documented behavioral features**,** n (%)†**	
Agitation	38 (42.2)
Physical aggression	37 (41.1)
Suicidal or homicidal ideation	31 (34.4)
Psychotic symptoms	14 (15.6)
**Agitation severity (OASS)**,** n (%)**	
Mild (0)	18 (20.0)
Moderate (1)	17 (18.9)
Severe (≥ 2)	55 (61.1)
**Alcohol withdrawal severity (CIWA-Ar)**, ***n* = 14**	
Mild	2 (14.3)
Moderate	6 (42.9)
Severe	6 (42.9)
**Opioid withdrawal severity (COWS)**, ***n* = 46**	
Mild	11 (23.9)
Moderate	19 (41.3)
Severe	16 (34.8)
**Initial pharmacologic regimen**,** n (%)**	
Antipsychotic-based regimen	80 (88.9)
Benzodiazepine-only regimen	10 (11.1)
**Adjunctive medication**,** n (%)**	
Biperiden‡	51 (56.7)
Pharmacologic escalation, n (%)	21 (23.3)
Psychiatric admission, n (%)	64 (71.1)

* Substance categories are non-mutually exclusive

† Behavioral features are non-mutually exclusive

‡ Biperiden was administered for extrapyramidal symptom prophylaxis or treatment and was not counted as pharmacologic escalation

Abbreviations: CIWA-Ar, Clinical Institute Withdrawal Assessment for Alcohol–Revised; COWS, Clinical Opiate Withdrawal Scale; IQR, interquartile range; OASS, Overt Agitation Severity Scale

### Behavioral phenotype and agitation severity

Behavioral features frequently overlapped: agitation in 42.2%, physical aggression in 41.1%, suicidal/homicidal ideation in 34.4%, and psychotic symptoms in 15.6% **(**Table 1**).**

Agitation severity was predominantly high, with severe agitation (OASS ≥ 2) in 61.1%, moderate in 18.9%, and mild in 20.0%.

Withdrawal scales were applied selectively. Among alcohol-only encounters (*n* = 14), CIWA-Ar was mostly moderate-to-severe, while among encounters with opioid involvement (*n* = 46), COWS was commonly moderate or severe **(**Table 1**)**. Because these scales were not applied uniformly, they were not included in multivariable modeling.

### Pharmacologic treatment and escalation

Initial pharmacologic strategies are summarized in Table 1 and Supplementary Table S1. Antipsychotic-based regimens predominated (88.9%), whereas benzodiazepine-only regimens were used in 11.1%. Biperiden was administered as an adjunct in 51 encounters, most commonly alongside antipsychotics.

Pharmacologic escalation occurred in 21 of 90 encounters (23.3%) and was defined as the addition of a second non-adjunctive antipsychotic or benzodiazepine agent during the same ED encounter. Escalation rates did not differ significantly by initial medication class or by primary substance category (Supplementary Table S2), suggesting that escalation patterns were not clearly differentiated by substance category or initial regimen choice in this cohort.

### Agitation severity and outcomes

Both escalation and psychiatric admission increased significantly across OASS strata **(**Table 2**)**:

**Table 2:** Pharmacologic escalation and psychiatric admission by OASS category

OASS category	Escalation *n*/*N* (%)	Admission *n*/*N* (%)
Mild (0)	0/18 (0.0)	9/18 (50.0)
Moderate (1)	4/17 (23.5)	10/17 (58.8)
Severe (≥ 2)	17/55 (30.9)	45/55 (81.8)
**Total**	21/90 (23.3)	64/90 (71.1)

Trend tests: escalation *p* = 0.012; psychiatric admission *p* = 0.016

Abbreviations: OASS, Overt Agitation Severity Scale

- Escalation: 0.0% (mild) → 23.5% (moderate) → 30.9% (severe); *p* = 0.012.
- Psychiatric admission: 50.0% → 58.8% → 81.8%; *p* = 0.016.

This graded pattern indicates that short-term ED outcomes varied across agitation severity strata.

### Substance involvement and outcomes

Across substance groupings (stimulant-related vs. alcohol-only vs. opioid-only), rates of severe agitation (*p* = 0.94), escalation (*p* = 0.65), and psychiatric admission (*p* = 0.18) did not differ significantly (Supplementary Table S2). Although stimulant-related encounters showed numerically higher admission than opioid-only presentations, substance category alone was not a reliable predictor of escalation or disposition.

### Multivariable predictors and discrimination

In multivariable logistic regression (Table 3), severe agitation (OASS ≥ 2) independently predicted psychiatric admission (aOR 7.87, 95% CI 2.28–27.17; *p* = 0.001), while increasing age was inversely associated with admission (aOR 0.91 per year, 95% CI 0.86–0.96; *p* < 0.001). Benzodiazepine-only regimen was not independently associated with disposition (*p* = 0.24). Model calibration was acceptable (Hosmer–Lemeshow *p* = 0.41; likelihood ratio *p* = 0.002).

**Table 3:** Multivariable logistic regression for psychiatric admission

Predictor	Crude OR (95% CI)	Adjusted OR (95% CI)	*p*-value	VIF
Severe agitation (OASS ≥ 2)	4.50 (2.10–9.65)	7.87 (2.28–27.17)	0.001	1.8
Age (per year increase)	0.94 (0.90–0.98)	0.91 (0.86–0.96)	< 0.001	1.2
Benzodiazepine-only regimen*	0.55 (0.15–2.10)	0.41 (0.09–1.83)	0.24	1.1

Model diagnostics: Hosmer–Lemeshow *p* = 0.41; optimism-corrected AUC = 0.76 (95% CI 0.66–0.85)

*Reference category: antipsychotic-based regimen

Abbreviations: OR, odds ratio; CI, confidence interval; OASS, Overt Agitation Severity Scale; VIF, variance inflation factor

Bootstrap internal validation of the age+OASS model yielded an optimism-corrected AUC of 0.76 (95% CI 0.66–0.85) for psychiatric admission. Exploratory ROC analysis identified OASS ≥ 2 as the optimal cut-point (sensitivity 81.8%, specificity 50.0%, PPV 81.8%, NPV 50.0%; LR + 1.64, LR − 0.36; Supplementary Table S3).

## Discussion

This study provides evidence that, in this single-center retrospective cohort of substance-related behavioral emergencies, observable agitation severity was associated with psychiatric admission and demonstrated a graded pattern of pharmacologic escalation across OASS strata. Although only psychiatric admission remained independently associated with agitation severity in multivariable analysis, pharmacologic escalation also increased progressively across OASS strata in unadjusted analyses. These findings suggest that observable behavioral severity may remain clinically relevant across diverse substance-related presentations encountered in the ED.

### Phenotype-first model in emergency agitation management

Current emergency psychiatry consensus, particularly Project BETA, prioritizes rapid behavioral control and safety over pinpointing specific causes [7, 8]. Yet much of the supporting evidence comes from psychiatric, mixed, or general ED agitation cohorts rather than substance-related ED populations [10, 19]. Our findings suggest that this paradigm may also be applicable to substance-related presentations, as agitation severity appeared to show closer associations with escalation and disposition outcomes than substance attribution within this cohort. The graded associations we observed—escalation rising from 0% to 30.9% and admission from 50% to 81.8%—illustrate this pattern within a substance-related ED cohort [7, 8, 14].

ED pharmacotherapy studies comparing commonly used agents have demonstrated differences in sedation profiles while emphasizing pragmatic agent selection based on safety, route feasibility, and clinical context [10, 14]. Our observational findings align with these trials: initial medication class was not independently associated with psychiatric admission, and escalation rates were comparable across regimens. These findings suggest that treatment intensification in routine care was more closely aligned with increasing agitation severity than with a fixed substance-based protocol; this is consistent with the pragmatic decision-making process in the emergency department, where the feasibility and safety of a treatment approach typically determine early treatment choices [8, 10].

Our findings align with three overlapping areas of literature: psychiatric agitation frameworks emphasizing behavioral control [7, 8], ED medication trials demonstrating broadly comparable short-term effectiveness across commonly used regimens [10, 14], and ED studies linking agitation severity to sedative or restraint use [13]. Taken together, these findings suggest that structured behavioral assessment may help guide early management decisions in substance-related behavioral emergencies.

### Substance-specific effects and the role of severity confounding

Prior ED studies have linked stimulant exposure to higher agitation scores and greater use of sedatives or restraints [13]. In our cohort, stimulant involvement was common and associated with a high prevalence of severe agitation (61.4%), comparable to previous series [13, 15]. This suggests that the association between stimulant exposure and adverse behavioral outcomes may be partly explained by baseline agitation severity [7, 8, 10].

Younger age independently predicted psychiatric admission, a pattern described in several ED behavioral cohorts [13, 15]. This may reflect clinicians’ concerns regarding impulsivity, environmental risk, or limited outpatient support, and underscores that the patient’s condition is shaped by both phenotype and systemic context. The independent effect of age alongside the OASS suggests that while observable behavioral severity plays an important role in short-term decisions, long-term risk assessment also accounts for demographic and social factors.

### OASS ≥ 2 as a pragmatic clinical threshold

We defined severe agitation as OASS ≥ 2, consistent with the original scale’s purpose. At this threshold, patients exhibit physical aggression or significantly impede care and require urgent intervention [12]. Practically, OASS ≥ 2 reflects the situation that emergency department clinicians define as “severe”; this is a point where safety risks increase, more staff are required, and the medical examination becomes difficult or unsafe. Other structured tools, such as the BARS, similarly aim to standardize the severity of observable agitation and support the broader clinical relevance of structured behavioral assessment [16].

ROC analysis showed that OASS ≥ 2 performed best for identifying psychiatric admission in this cohort (sensitivity 81.8%, LR − 0.36). Specificity was lower, likely because most patients in the cohort were ultimately admitted. In practice, patients with OASS ≥ 2 generally represented the group requiring closer monitoring and more intensive behavioral management, although lower scores did not rule out admission.

The combination of age and OASS produced an optimism-corrected AUC of 0.76 after bootstrap validation. This level of discrimination was achieved without depending on toxicology confirmation or precise substance identification, which are often unavailable during the first stages of ED evaluation. Our findings therefore support the idea that observable agitation severity may still provide clinically useful information early in the course of care [7, 8].

### Clinical implications and implementation

Our findings may have practical implications for emergency department care and resource management. Routine incorporation of OASS or similar observational scales into ED assessment workflows for agitated patients may improve documentation, facilitate communication among team members, and trigger predefined care protocols when threshold values such as ≥ 2 are reached. Embedding OASS fields and automated alerts into ED information systems may standardize triage and reduce practice variation. Second, using severity-based pathways in quality improvement work could help EDs monitor sedative use, restraint practices, and boarding times in a more reproducible way, independent of diagnostic uncertainty about substance attribution. Third, focusing on phenotype may improve staff safety. High-risk patients may be identified from observable behavior while collateral history, toxicology results, and substance-specific assessment are still being obtained. Clinically, this supports the use of behavior-informed protocols during initial stabilization, while leaving room for substance-specific assessment and treatment. After the patient is calm enough to assess safely, substance-specific management, including withdrawal protocols and targeted pharmacotherapy, can be incorporated [8, 10]. We do not suggest that substance-specific assessment or toxidrome recognition is unimportant. Rather, our findings indicate that when diagnostic uncertainty is common, observable behavioral severity may still provide clinically useful guidance during the early stabilization phase, while substance-specific management remains essential once a more complete assessment becomes possible.

### Limitations

This study has several limitations. First, the retrospective single-center design limits causal inference and may restrict generalizability to EDs with different patient populations, organizational structures, psychiatric consultation availability, or substance-use patterns. Substance classification and exclusion of non-psychiatric medical etiologies were based on retrospective clinical assessment and documentation rather than standardized confirmatory testing, which may have introduced misclassification and selection bias, particularly in patients with overlapping causes of behavioral disturbance.

Second, as with all retrospective record reviews, incomplete documentation may have affected data quality. No data imputation was performed, and encounters with insufficient documentation were excluded. OASS scores were retrospectively derived from clinical documentation rather than prospectively assigned in real time, introducing potential measurement bias despite high inter-rater reliability. In addition, collapsing OASS scores 2–6 into a single severe category may have reduced granularity among higher-severity presentations.

Third, withdrawal scales such as CIWA-Ar and COWS were applied selectively and could not be modeled as independent predictors. Inclusion was limited to patients requiring pharmacologic behavioral control, and the analytic cohort should therefore be interpreted as a selected subgroup rather than representative of all ED patients with substance-related agitation. Furthermore, with only 90 encounters across approximately 200,000 annual visits, provider experience with this patient subgroup may have varied, potentially contributing to heterogeneous management decisions. Finally, pharmacologic escalation excluded biperiden use and dose titration of the same agent, and did not capture non-pharmacologic de-escalation, physical restraint use, or clinician intent. Model performance was assessed using internal validation only; external validation in larger multicenter cohorts is required before clinical implementation.

## Conclusion

In substance-related behavioral emergencies, agitation severity—not substance category or initial medication class—was independently associated with psychiatric admission in this cohort and showed a graded association with pharmacologic escalation across OASS strata in unadjusted analyses. Short-term outcomes varied across OASS strata in our cohort. A simple model including age and agitation severity showed clinically meaningful discrimination for psychiatric admission. These findings suggest that structured behavioral assessment may help guide early ED management in substance-related behavioral emergencies. During acute stabilization, observable behavioral severity may still provide clinically useful guidance before toxicology results or definitive etiologic clarification are available. The routine use of structured agitation scales such as OASS may improve the triage of emergency department resources and patient referral planning. Future multicenter studies are needed to validate severity-based triage approaches and evaluate their impact on safety and patient-centered outcomes.

## Supplementary Information

Below is the link to the electronic supplementary material.

Supplementary Material 1

## Data Availability

The datasets used and/or analysed during the current study are available from the corresponding author on reasonable request.

## Abbreviations

AUC: Area under the receiver operating characteristic curve
CI: Confidence interval
CIWA: Ar–clinical institute withdrawal assessment for alcohol–revised
COWS: Clinical opiate withdrawal scale
ED: Emergency department
IQR: Interquartile range
LR+: Positive likelihood ratio
LR–: Negative likelihood ratio
OASS: Overt agitation severity scale
OR: Odds ratio
ROC: Receiver operating characteristic
VIF: Variance inflation factor
