# Supplementary material for: Agitation severity and emergency department outcomes in substance-related behavioral emergencies: a retrospective cohort study
Source: BMC Emerg Med. 2026 Jun 18;26:239. doi: 10.1186/s12873-026-01646-0 (PMC13548589; doi:10.1186/s12873-026-01646-0)
Supplement: Supplementary file 1 [file 12873_2026_1646_MOESM1_ESM.docx]

**Supplementary Table S1. Initial pharmacologic regimen according to agitation severity and substance-related presentation**

| Variable | Antipsychotic-based  (n = 80) | Benzodiazepine-only  (n = 10) | p-value |
| --- | --- | --- | --- |
| Age, years, median (IQR) | 31 (25–38) | 35 (30–40) | 0.09 |
| Severe agitation (OASS ≥2), n (%) | 47 (58.8) | 8 (80.0) | 0.25 |
| Stimulant-related presentation†, n (%) | 60 (75.0) | 8 (80.0) | 0.74 |

***p-values obtained using Mann–Whitney U test for continuous variables and χ² or Fisher’s exact test for categorical variables.
†Stimulant-related presentations include stimulant-only and stimulant-containing polysubstance use.***

***Abbreviations:*** *OASS, Overt Agitation Severity Scale.*

**Supplementary Table S2. Clinical features and outcomes according to substance-related presentation**

| Variable | Stimulant-related*  (n = 70) | Alcohol-only  (n = 14) | Opioid-only  (n = 6) | p-value |
| --- | --- | --- | --- | --- |
| Severe agitation (OASS ≥2), n (%) | 43 (61.4) | 8 (57.1) | 4 (66.7) | 0.94 |
| Physical aggression, n (%) | 30 (42.9) | 5 (35.7) | 2 (33.3) | 0.88 |
| Psychotic features, n (%) | 15 (21.4) | 2 (14.3) | 0 (0.0) | 0.32 |
| Pharmacologic escalation, n (%) | 17 (24.3) | 2 (14.3) | 2 (33.3) | 0.65 |
| Psychiatric admission, n (%) | 53 (75.7) | 9 (64.3) | 2 (33.3) | 0.18 |

****Stimulant-related includes stimulant-only and stimulant-containing polysubstance presentations. Percentages reflect column percentages.***

*p-values obtained using χ² or Fisher’s exact test as appropriate.*

***Abbreviations:*** *OASS, Overt Agitation Severity Scale.*

**Supplementary Table S3. Diagnostic performance of OASS ≥2 threshold for predicting psychiatric admission**

| ****Metric**** | ****OASS ≥2**** |
| --- | --- |
| Sensitivity | **81.8%** |
| Specificity | **50.0%** |
| PPV | **81.8%** |
| NPV | **50.0%** |
| LR+ | **1.64** |
| LR- | **0.36** |
| Youden Index | **0.32** |
| AUC (95% CI) | **0.76 (0.66–0.85)** |

***Derived from ROC analysis of raw OASS (0–6) for prediction of psychiatric admission; optimism-corrected using 1,000 bootstrap resamples.***

***Abbreviations:*** *OASS, Overt Agitation Severity Scale; AUC, area under the curve; CI, confidence interval; LR, likelihood ratio.*
